# Supplementary material for: Spatial Variation in Stream-Water Bacterial Communities Across Sites Differing in Wild Chinese Giant Salamander Detection Frequency
Source: Biology (Basel). 2026 Sep 18;15(18):1647. doi: 10.3390/biology15181647 (PMC13604818; doi:10.3390/biology15181647)
Supplement: Supplementary file 1 [file biology-15-01647-s001.zip › Data Related to “Spatial Variation in Stream-Water Bacterial Communities across Sites Differing in Wild Chinese Giant Salamander Detection Frequency”/Microbial Data/report/src/hyperlinks/Methods_QIIME2_EN_LRS.pdf]

Method

The method is only used as a reference for publication purpose. Customers are responsible for the related risks of duplicate checking.

1 Experimental Procedure

1.1 Genomic DNA Extraction and Quality Assessment

Genomic DNA was extracted according to sample type. The extracted DNA was then subjected to quality assessment via 1% agarose gel electrophoresis (120V, 30 min) to evaluate band integrity and nucleic acid purity. Qualified DNA samples were diluted with sterile nuclease-free water to a final concentration of 1 ng/μL for downstream experiments.

1.2 PCR Amplification and Product Purification

The diluted genomic DNA was used as the template for PCR amplification of the target 16S rRNA gene region using barcode-tagged primers. PCR was performed in a reaction containing 12.5 μL KAPA HiFi HotStart ReadyMix, 2.5 μM primers, and 10 ng genomic DNA template. The thermal cycling conditions were as follows: initial denaturation at 95℃ for 3 min, followed by 22 cycles of denaturation at 98℃ for 20 s, annealing at 57℃ for 30 s, and extension at 72℃ for 75 s, with a final extension at 72℃ for 5 min. The PCR products were verified by 2% agarose gel electrophoresis and purified using magnetic bead- based purification kits (e.g., AMPure XP) to remove primer dimers.

1.3 Kinnex Library Preparation

Barcoded PCR products from different samples were pooled in equimolar amounts, and the Kinnex long-read sequencing technology was employed for library preparation. The detailed steps are as follows:

End Repair: PCR products were treated with Kinnex End Repair Enzyme (20°C, 30 min) to ensure blunt-ended DNA fragments.

Adapter Ligation: Kinnex-specific sequencing adapters (20 μM) and T4 DNA ligase (20°C, 15 min) were added to ligate DNA fragments to sequencing adapters.

Linear Array Assembly: The 16S rRNA gene fragments were concatenated into long linear DNA arrays using Kinnex Assembly Enzyme (37°C, 1 h).

Library Purification: SMRTbell® magnetic beads were used for size selection (0.45× and 0.8× bead ratios) to retain target fragments ranging from 500 to 2000 bp, thereby improving library quality.

1.4 Library Quality Evaluation and Sequencing

The purified library was quantified using a Qubit® 4.0 Fluorometer, and its size distribution was assessed using an Agilent 2100 Bioanalyzer to ensure the primary peak fell within the expected range (1.5–3 kb). Libraries meeting quality standards were sequenced on the Revio platform using Single Molecule, Real-Time (SMRT) sequencing to obtain high-quality long-read data.

2 Bioinformatics Analysis Pipeline

1.1 LongReads Quality Control

Quality filtering on the raw tags were performed using the dada2 software to obtain high-quality Clean Tags.

1.2 ASVs Denoise and Species Annotation

1.2.1 ASVs Denoise

For the Effective Tags obtained previously, denoise was performed with DADA2 or deblur module in the QIIME2 software to obtain initial ASVs (Amplicon Sequence Variants) (default: DADA2).

1.2.2 Species Annotation

16S: For each representative sequence, the Silva Database (http://www.arb- silva.de/) (Quast C et al.,2012) was used based on Mothur algorithm to annotate taxonomic information.

18S: For each representative sequence, the Silva Database (http://www.arb- silva.de/) (Quast C et al.,2012) was used based on RDP classifier algorithm to annotate taxonomic information.

ITS: For each representative sequence, the Unite Database (https://unite.ut.ee/) (Herr JR et al.,2014) was used based on blast algorithm to annotate taxonomic information.

Unregular region: Micro_NT by default (a sub library obtained by extracting archaea, fungi, viruses, and bacteria from the NT) .

Note:

1. The fasta file (SILVA_138.1_SSURef_NR99_tax_silva.fasta) from the Silva offical w ebsite only provide species name rather than taxnonomic information, and thier taxonomic in formation file is incomplete. Therefore, we need use species name to get their taxonomic inf ormation from NCBI. At first, taxonomic information file from Silva offical is used to annotate, for those sequences that can not be annotated, a dmp file provided by NCBI would be use t o supplement the taxonomic information.

2. Annotation principle of Micro_NT: perform sequence alignment (BLAST) between our sequence and sequence in Micro_NT. Then obtain the top20 result according to the score, s creen by the maximum bit-score, use LCA algorithm to infer the Lowest Common Ancestor. Due to the large amount of unclassified information in the NT, in order to ensure the accuracy of the data, the annotation effect is greatly reduced, resulting in more unclassified results. So when we use LCA algorithm to obtain species information, we will ignore unclassified species to reduce their impact on annotation.

1.2.3 Phylogenetic Relationship Construction

In order to study phylogenetic relationship of different ASVs, and the difference of the dominant species in different samples (groups), multiple sequence alignment were conducted using the MUSCLE software (Version 3.8.31， http://www.drive5.com/muscle/) (Edgar RC et al.,2004).

1.2.4 Data Normalization

ASVs abundance information were normalized using a standard of sequence number corresponding to the sample with the least sequences. Subsequent analysis of alpha diversity and beta diversity were all performed basing on this output normalized data.

1.2.5 Relative abundance

Top 10 taxa of each samples at each taxonomic ranks(Phylum, Class, Order, Family, Genus, Species) were selected to plot the distribution histogram of relative abundance in Perl through SVG function.

1.2.6 Heatmap

The abundance information of top 35 taxa of each samples at each taxonomic ranks were used to drawn the heatmap, which visually display different abundance and taxa clustering. This was achieved in R through the pheatmap() function.

1.2.7 Ternary plot

Ternary plot for top 10 taxa at each taxonomic ranks can be used to show the abundance difference among three samples. It was performed in R with vcd() function.

1.2.8 Venn and Flower diagram

Venn and Flower diagrams visually display the common and unique information between different samples or groups. Venn and Flower diagrams were produced in R with VennDiagram() function and in perl with SVG function, respectively.

1.2.9 Phylogenetic tree

Phylogenetic tree, also called evolutionary tree, can describe the evolutionary relationship between different species. One hundred genera with the highest abundance in the samples were selected and performed sequence alignment to draw the phylogenetic tree in perl with SVG function.

1.3 Alpha Diversity

1.3.1 Alpha Diversity

Alpha diversity is applied in analyzing complexity of species diversity for a sample through 6 indices, including Observed-species, Chao1, Shannon, Simpson, ACE, Good- coverage. All this indices in our samples were calculated with QIIME (Version 1.9.1) and displayed with R software (Version 4.0.3).

Two indices were selected to identify Community richness:

Chao -the Chao1 estimator (http://www.mothur.org/wiki/Chao); CE -the ACE estimator (http://www.mothur.org/wiki/Ace);

Two indices were used to identify Community diversity:

Shannon - the Shannon index (http://www.mothur.org/wiki/Shannon); Simpson - the Simpson index (http://www.mothur.org/wiki/Simpson); One indice to characterized Sequencing depth:

Coverage - the Good’s coverage (http://www.mothur.org/wiki/Coverage)

1.3.2 Species accumulation boxplot

In order to evaluate the richness of microbial community and sample size. Species accumulation boxplot can be used to visualize, which performed with vegan package in R software.

1.3.3 Rank abundance curve

Rank abundance curve can reflect the richness and evenness of species with samples through observing the width and shape of the curves. It can be plotted by using the RColorBrewer package in R.

1.3.4 Rarefaction curve

Insufficient sequencing number may lead to insufficient sample information, whereas excessive sequencing rounds can also result in unnecessary increase in cost. Therefore, the determination for the proper amount of sequencing is essential. Plotting rarefaction curves provides the ability to discover the viability of the sequencing numbers. This is achieved through R with the plyr library.

1.4 Beta Diversity

1.4.1 Beta diversity

Beta diversity analysis was used to evaluate differences of samples in species complexity, Beta diversity on both weighted and unweighted unifrac were calculated by QIIME software (Version 1.9.1).

Cluster analysis was preceded by principal component analysis (PCA), which was applied to reduce the dimension of the original variables using the ade4 package and ggplot2 package in R software(Version 4.0.3).

Principal Coordinate Analysis (PCoA) was performed to get principal coordinates and visualize from complex, multidimensional data. A distance matrix of weighted or unweighted unifrac among samples obtained before was transformed to a new set of orthogonal axes, by which the maximum variation factor is demonstrated by first principal coordinate, and the second maximum one by the second principal coordinate, and so on. PCoA analysis was displayed by ade4 package and ggplot2 package in R software(Version 4.0.3).

1.4.2 Beta diversity heatmap

Beta diversity analysis was used to evaluate differences of samples in species complexity. Beta diversity on both weighted and unweighted unifrac were calculated by QIIME software. Then a heatmap was created to display the unifrac distance between samples, which was realized in Perl.

1.4.3 Unweighted Pair-group Method with Arithmetic Mean (UPGMA)

A cluster tree is constructed on UPGMA, which is based on the weighted unifrac distance matrix. This is widely used in ecology for evolutionary classification. UPGMA diagram was drawn through the upgma.tre function within Qiime.

1.4.4 Cluster analysis

Cluster analysis was preceded by principal component analysis (PCA), which was applied to reduce the dimension of the original variables using the ade4 package and ggplot2 package with R software(Version 4.0.3).

Principal Coordinate Analysis (PCoA) was performed to get principal coordinates and visualize from complex and multidimensional data. A distance matrix of weighted or unweighted unifrac among samples was obtained before transformation to a new set of orthogonal axes, by which the maximum variation factor is demonstrated by first principal coordinate, and the second maximum one by the second principal coordinate, and so on. PCoA analysis was displayed by ade4 package and ggplot2 package in R software(Version 4.0.3).

Non-metric multidimensional scaling (NMDS) was also implemented for data dimension reduction. Similar to the PCoA, NMDS also use the distance matrix but it emphasizes the numerical rank instead. The distance between sample points on the diagram can only reflect the rank information rather than the numerical differences. NMDS analysis was implemented through R software with ade4 package and ggplot2 package.

1.5 Community difference analysis

A series of statistical analyses which inlcude Anosim, Adonis, Multi-response permutation procedure (MRPP), Simper, T-test, MetagenomeSeq and LEfSe, were performed to reveal the community structure differentiation.

Anosim, Adonis and MRPP analysis are non-parametric tests that analyze the difference between high-dimensional data group. They can test whether the differences between groups are significant greater than the differences within the group, which can determine whether the grouping is meaningful. All of them were performed with vegan package and ggplot2 package within R.

The Wilcoxon rank-sum test (also known as the Mann–Whitney U test) is a nonparametric statistical method for comparing two independent samples. The null hypothesis states that the two populations from which the samples are drawn exhibit identical distributions. By analyzing the average ranks of the samples, this test determines whether the distributions differ significantly. It can be applied to assess differences in species composition between groups.

The Kruskal-Wallis H test (Kruskal-Wallis rank-sum test) is a nonparametric method that extends the two-sample Wilcoxon rank-sum test to multiple independent samples (⩾3 groups). This analysis can assess significant differences in species composition across multiple sample groups.

Simper can reveal the contribution of each species to the differentiation between groups. Top 10 species were selected and presented on the graph. It was performed in R with Vegan package and ggplot2 package.

MetagenomeSeq can showcase the species that display significant differences between groups. It was performed in R with metagenomeSeq package.

LEfSe is widely used to discover biomarkers and it can reveal metagenomic characteristics. To achieve this, a exclusive package named lefse was utilized.

1.6 Function prediction

PICRUSt (V1.1.4) is a package in R and is mainly used to predict the metagenomic functions based on marker genes. PICRUSt2 (V2.3.0) is the improved version of PICRUSt.

Tax4Fun (V0.3.1) is a R package that is widely used for intestinal and soil samples. In general, it can supply more accurate results when compared with PICRUSt, especially for soil samples.

BugBase is an excellent tool to discover the phenyotpe of microogranisms. It can classify the microbial communities according to seven phenotypes: Gram Positive, Gram Negative, Biofilm Forming, Pathogenic, Mobile Element Containing, Oxygen Utilization, including Aerobic, Anaerobic, and Cultivable Anaerobic, and Oxidative Stress Tolerance.

FunGuild is an excellent tool through python when working with fungi samples.

FAPROTAX through python can play a great role when elucidating the possible biochemical processes and elements in play.

1.7 Association analysis

To explore the symbiotic relationship between species and to reveal the environmental factor influence on community structures, 2D and 3D network diagrams were drew for

visualization. Further analyses such as spearman correlation test, canonical correspondence analysis (CCA)/redundancy analysis (RDA) and dbRDA can be used to reflect the correlation between environmental factors and species abundance. All of these diagram and analysis were completed in R.

1.8 Community Assembly Mechanisms

To investigate the influence of environmental factors on microbial community assembly processes, we employed the Beta Nearest Taxon Index (βNTI) analysis. βNTI is a phylogenetic β-diversity metric that quantifies the degree of divergence between the most phylogenetically related taxa (nearest taxon) across samples. Specifically, βNTI compares the observed phylogenetic turnover (βMNTD, beta mean nearest taxon distance) to null model expectations, thereby distinguishing whether community differences are primarily driven by stochastic processes or deterministic selection.

2 Reference

Bokulich NA, Subramanian S, Faith JJ, et al. Quality-filtering vastly improves diversity estimates from Illumina amplicon sequencing.Nature Methods. 2012;10(1):57-59. doi:10.1038/nmeth.2276.

Edgar RC, Haas BJ, Clemente JC, Quince C, Knight R. UCHIME improves sensitivity and speed of chimera detection.Bioinformatics. 2011;27(16):2194-2200. doi:10.1093/bioinformatics/btr381.

Edgar RC. MUSCLE: multiple sequence alignment with high accuracy and high throughput.Nucleic Acids Research. 2004;32(5):1792-1797. doi:10.1093/nar/gkh340.

Edgar RC. UPARSE: highly accurate OTU sequences from microbial amplicon reads.Nature Methods. 2013;10(10):996-998. doi:10.1038/nmeth.2604.

Herr JR, Öpik M, Hibbett DS. Towards the unification of sequence-based classification and sequence-based identification of host-associated microorganisms.New Phytologist. 2014;205(1):27-31. doi:10.1111/nph.13180.

Magoc T, Salzberg SL. FLASH: fast length adjustment of short reads to improve genome assemblies.Bioinformatics. 2011;27(21):2957-2963. doi:10.1093/bioinformatics/btr507.

Quast C, Pruesse E, Yilmaz P, et al. The SILVA ribosomal RNA gene database project: improved data processing and web-based tools.Nucleic Acids Research. 2012;41(D1):D590-D596. doi:10.1093/nar/gks1219.
